# Supplementary material for: The Association of Physical (in)Activity with Mental Health. Differences between Elder and Younger Populations: A Systematic Literature Review
Source: Int J Environ Res Public Health. 2021 Apr 29;18(9):4771. doi: 10.3390/ijerph18094771 (PMC8124550; doi:10.3390/ijerph18094771)
Supplement: Supplementary file 1 [file ijerph-18-04771-s001.zip › Supplementary Material - Summary table/Search startegy EconLit.pdf]

Friday, December 11, 2020 7:05:35 AM

#	Query	Limiters/Expanders	Last Run Via	Results
S12	((S1 OR S2) AND (S3 OR S4) AND (S5 OR S6)) NOT (S7 OR S8)	Limiters - Linked Full Text; Published Date: 20000101-20201231<br>Search modes - Find all my search terms	Interface - EBSCOhost Research Databases<br>Search Screen - Advanced Search<br>Database - EconLit	13
S11	((S1 OR S2) AND (S3 OR S4) AND (S5 OR S6)) NOT (S7 OR S8)	Limiters - Published Date: 20000101-20201231<br>Search modes - Find all my search terms	Interface - EBSCOhost Research Databases<br>Search Screen - Advanced Search<br>Database - EconLit	51
S10	((S1 OR S2) AND (S3 OR S4) AND (S5 OR S6)) NOT (S7 OR S8)	Expanders - Apply equivalent subjects<br>Search modes - Find all my search terms	Interface - EBSCOhost Research Databases<br>Search Screen - Advanced Search<br>Database - EconLit	53
S9	(AB (descriptive analys* OR ANOVA OR correlations measures)) AND (S1 AND S3 AND S5)	Expanders - Apply equivalent subjects<br>Search modes - Find all my search terms	Interface - EBSCOhost Research Databases<br>Search Screen - Advanced Search<br>Database - EconLit	0
S8	AB (descriptive analys* OR ANOVA OR correlations measures)	Expanders - Apply equivalent subjects<br>Search modes - Find all my search terms	Interface - EBSCOhost Research Databases<br>Search Screen - Advanced Search<br>Database - EconLit	7,238
S7	TI (descriptive analys* OR ANOVA OR correlations measures)	Expanders - Apply equivalent subjects<br>Search modes - Find all my search terms	Interface - EBSCOhost Research Databases<br>Search Screen - Advanced Search<br>Database - EconLit	170
S6	AB Quantitative studies OR quantitative analys* OR regression OR econometric* OR association OR cross-section* OR longitudinal analys* OR panel data analys* OR causality*	Expanders - Apply equivalent subjects<br>Search modes - Find all my search terms	Interface - EBSCOhost Research Databases<br>Search Screen - Advanced Search<br>Database - EconLit	118,129

S5	TI Quantitative studies OR quantitative analys* OR regression OR econometric* OR association OR cross-section* OR longitudinal analys* OR panel data analys* OR causality*	Expanders - Apply equivalent subjects<br>Search modes - Find all my search terms	Interface - EBSCOhost Research Databases<br>Search Screen - Advanced Search<br>Database - EconLit	26,302
S4	AB Physical activity OR physical inactivity OR Physical exertion OR exercise OR physical exercise OR sport OR physical education	Expanders - Apply equivalent subjects<br>Search modes - Find all my search terms	Interface - EBSCOhost Research Databases<br>Search Screen - Advanced Search<br>Database - EconLit	14,767
S3	TI Physical activity OR physical inactivity OR Physical exertion OR exercise OR physical exercise OR sport OR physical education	Expanders - Apply equivalent subjects<br>Search modes - Find all my search terms	Interface - EBSCOhost Research Databases<br>Search Screen - Advanced Search<br>Database - EconLit	2,623
S2	AB Mental health OR Mental disorder* OR depress* OR anxiety OR psychiatr*	Expanders - Apply equivalent subjects<br>Search modes - Find all my search terms	Interface - EBSCOhost Research Databases<br>Search Screen - Advanced Search<br>Database - EconLit	8,225
S1	TI Mental health OR Mental disorder* OR depress* OR anxiety OR psychiatr*	Expanders - Apply equivalent subjects<br>Search modes - Find all my search terms	Interface - EBSCOhost Research Databases<br>Search Screen - Advanced Search<br>Database - EconLit	3,205
